# Development and Validation of a Caregiving Knowledge Questionnaire for Parents of Pediatric Leukemia and Lymphoma Patients in Malaysia

**DOI:** 10.7759/cureus.30903

**Published:** 2022-10-31

**Authors:** Chai-Eng Tan, Sie Chong Doris Lau, Kit Aun Tan, Zarina Abdul Latiff, Kok Hoi Teh, Chee Chan Lee, Sherina Mohd Sidik

**Affiliations:** 1 Department of Family Medicine, Faculty of Medicine, Universiti Kebangsaan Malaysia, Kuala Lumpur, MYS; 2 Department of Psychiatry, Universiti Putra Malaysia, Serdang, MYS; 3 Department of Pediatrics, Faculty of Medicine, Universiti Kebangsaan Malaysia, Kuala Lumpur, MYS; 4 Department of Pediatrics, Hospital Tunku Azizah (Ministry of Health Malaysia), Kuala Lumpur, MYS

**Keywords:** malaysia, questionnaire development and validation, parents’ knowledge, primary caregiver, pediatric leukemia and lymphoma

## Abstract

Introduction

Validated tools to measure caregiving knowledge among parents of children with hematological cancers are needed to measure the clinical outcome of caregiver interventions. This study reports the development and validation of the Hematological Oncology Parents Education Caregiving Knowledge Questionnaire (HOPE-CKQ) among Malaysian parents of pediatric leukemia and lymphoma patients.

Methods

Initially, 60 items on caregiving knowledge were developed based on a qualitative needs assessment study. Content validity was evaluated using item content validity index (I-CVI) and scale content validity index (S-CVI/Ave). Parents of pediatric leukemia and lymphoma patients were invited to complete the 60-item version of the HOPE-CKQ. Exploratory factor analysis (EFA) using polychoric correlation resulted in an 18-item version of HOPE-CKQ. Confirmatory factor analysis (CFA) was used to verify the factor structure. Known-group validity was tested by comparing the scores among different levels of parent education.

Results

The I-CVI ranged from 0.83 to 1.00 whereas the S-CVI/Ave was 0.99, indicating good content validity. A total of 167 complete responses were analyzed for factor analysis. EFA using polychoric correlations resulted in a single-factor structure consisting of 18 items. CFA confirmed that the 18-item single-factor HOPE-CKQ model had a good fit for the data. The internal consistency reliability was good (*α*=0.80). Parents with tertiary education level had higher caregiving knowledge (M=12.61, SD=3.37) compared to parents with secondary education and below (M=10.33, SD=3.80) (t=3.58, p<0.001).

Conclusions

The 18-item HOPE-CKQ is valid and reliable for use to measure caregiving knowledge among pediatric leukemia and lymphoma parents. This tool may be considered to measure caregiving knowledge in future preventive and intervention programs.

## Introduction

The diagnosis of cancer in a child impacts the whole family. Parents play the pivotal role of caregiver while balancing the family’s needs as well as job demands [1]. Their caregiving role encompasses medical caregiving and decision-making, providing emotional support to the child and family members, and ensuring the basic needs of the family members are met [1,2]. Providing psychosocial support to parents to aid them in their caregiving tasks may reduce their psychological distress and indirectly impact the child’s psychological well-being as well [3].

A major component of psychosocial care for parents is psychoeducation, defined as “the structured provision of information regarding the disease process, resources and services, as well as caregiver training to enable effective response to the disease-related problems” [4]. Psychoeducational interventions may include health education, training on problem-solving or coping skills, and psychological interventions, such as cognitive behavior therapy or provision of social support [5]. These interventions have shown good results on psychological outcomes among parents of children with cancer [3,5]. Caregiver education is one of the simplest, yet most important forms of psychoeducation.

Caregiver education should meet the various information needs of parents of children with cancer. These needs include information regarding the disease and treatment, how to provide care for the child at home, how to support the child emotionally, and how to access practical support and perform self-care [6,7]. The Children’s Oncology Group has proposed an educational checklist for parents of children who are newly diagnosed with cancer, which covers main topics of diagnosis and treatment, coping strategies and managing life demands, and care for the child [8,9]. Knowledge of these educational topics is important to allow parents to make sense of their child’s condition, to make medical decisions, and to play their roles as caregivers to their child.

Parents’ knowledge about appropriate home caregiving influences their caregiving practices for the child [10]. However, parents may have inadequate caregiving knowledge including infection prevention, managing side effects of treatment, and providing emotional support for their child [11]. Therefore, caregivers’ knowledge in these areas needs to be evaluated.

Despite the central role of parent or caregiver knowledge in the management of children with cancer, there were no validated questionnaires available to measure the level of caregiving knowledge. Past studies that measured parents’ caregiving knowledge did not use tools that have been validated [10,12-14]. There was insufficient description of how these tools were developed. In general, the tools tested knowledge regarding childhood leukemia or childhood cancer, general knowledge regarding its treatment, and effects of treatment. However, the tools were not adequately evaluated for their content and construct validity.

A reliable and valid tool is required to measure parents’ caregiving knowledge to evaluate the effectiveness of an educational program or to study factors related to caregiving knowledge. The items of the tool should adequately represent the domain of caregiving knowledge, for it to possess content validity [15]. Internal consistency reliability reflects the interrelatedness among the items [15]. In a multi-item tool, construct validity is needed to identify its dimensionality (structural validity) and whether the scores of the tool are consistent with relevant hypotheses [15].

Developing such a general tool for childhood cancer, in general, would be challenging in view of the variety of diagnosis and treatment-related knowledge, and the necessary core caregiving knowledge for individual cancer types. Hence, such a tool should be specific to certain cancer diagnoses. Hematological cancers such as leukemia and lymphoma form the bulk of childhood cancers. The Global Burden of Disease Study 2019 estimated about 657,563 cases of childhood leukemia, 26,443 cases of Hodgkin's lymphoma, and 26,551 cases of non-Hodgkin’s lymphoma among children aged 14 years and below [16]. Therefore, the decision was made to focus on the development of a new caregiving knowledge tool for pediatric leukemia and lymphoma parents.

This study describes the development and validation (structural validity, reliability, and known-group validity) of the Hematological Oncology Parents Education Caregiving Knowledge Questionnaire (HOPE-CKQ), to measure parents’ caregiving knowledge for children with leukemia and lymphoma.

## Materials and methods

There were two main phases of this study - (1) the development of the HOPE-CKQ and (2) the psychometric validation of the HOPE-CKQ.

Phase 1: the development and content validity of HOPE-CKQ

The HOPE-CKQ was developed based on a prior qualitative study that explored the information needs of parents of children with cancer [6]. A total of 60 true-false items were generated. These items encompass domains, such as disease and treatment-related knowledge, care for the child at home, and psychosocial support for the child. All items were in the Malay language, the national language of Malaysia, which is also spoken in Singapore, Indonesia, and Brunei.

These items were sent to an expert panel comprising two pediatricians, two primary care physicians, a nurse, and a parent of a child with lymphoma. The expert panel was asked to evaluate the items based on the relevance of the concept tested and the accuracy of the answers. They were also invited to provide open-ended feedback. The item content validity index (I-CVI) was determined from the proportion of evaluators who rated the item as relevant over the total number of evaluators. The scale content validity index (S-CVI/Ave) was determined by averaging the I-CVI for all items. The item was retained if the I-CVI was 0.80 and above [17]. Good content validity is indicated by I-CVI of at least 0.78 and S-CVI/Ave of at least 0.90 when there are six to 10 raters [17]. Open-ended feedback was also taken into consideration for the decision to replace or revise the item. Only five items needed modifications and they were further reviewed by the expert panel.

Phase 2: the psychometric validation of HOPE-CKQ

A cross-sectional validation study was conducted among parents of children with leukemia and lymphoma.

Participants

Parents were included if their child was diagnosed with leukemia or lymphoma at less than 18 years of age. The child could be receiving treatment or had completed treatment during the data collection period. We excluded other relatives who were not parents, parents who were not the primary caregiver of the child during treatment, parents who were experiencing extreme psychological distress, and parents who were not able to read or understand written Malay.

Sample Size Determination

Based on an estimated ratio of 12 variables to each factor and up to four factors extracted, a sample size of 180 parents was needed [18]. Kaiser-Meyer-Olkin measure of sampling adequacy and Bartlett’s test of sphericity were used to reconfirm the adequacy of sample size [19].

Data Collection

Data were collected via both online survey and face-to-face at two major pediatric oncology centers in Malaysia from October 2021 to February 2022. Both online survey and face-to-face methods were used to improve participation rates. Participants were invited to join the online survey via advertisements on social media and through the local cancer parent support groups. For face-to-face data collection, pediatric patients with leukemia and lymphoma were identified at the pediatric oncology clinic and wards. The parents who were accompanying the patients were invited to participate in the study and provided written informed consent. They completed the self-administered research questionnaire on the spot and returned it immediately to the researcher.

Measures

The research questionnaire contained the sociodemographic characteristics of the parent (age, gender, ethnicity, formal education status, and household income status), clinical characteristics of the child (diagnosis, age at diagnosis, duration of diagnosis, treatment phase, and treatment center), and the 60-item HOPE-CKQ. The questionnaire was administered in both online and face-to-face formats. Parents were to indicate if the HOPE-CKQ items were true, or false, or if they did not know the answers. This response format was presented in this way so that parents were not forced to guess if they did not know the answer. Each item received a score of 1 for correct answer and 0 for incorrect answer or if the parents indicated that they did not know the answer.

Data Analysis

As all HOPE-CKQ items had binary scores, exploratory factor analysis (EFA) with a polychoric correlation matrix was used to uncover the underlying structure of the 60-item HOPE-CKQ. Polychoric correlation is an extension of tetrachoric correlation, which measures the relationship between two binary variables [20]. EFA using the polychoric correlation matrix is more robust for scales with ordinal or binary responses which often do not possess multivariate normality [19,21].

Prior to EFA, all HOPE-CKQ items were examined for univariate normality and linearity. Items with skewness indices between -3 and +3, and kurtosis indices between -7 and +7 were indicative of normality [19]. As for the linearity assumption, inter-item correlation coefficients should range between 0.30 and 0.80 [22]. Items that did not meet the criteria for univariate normality and linearity were removed from subsequent analysis.

EFA was conducted using FACTOR software (Tarragona, Spain: Universitat Rovira i Virgili) [21,23]. Parallel analysis using minimum rank factor analysis was used to determine the number of factors to be retained. Factor extraction was performed using the unweighted least squares method. The unweighted least squares method is more robust for standard errors, does not depend on the distribution of observed variables [24], and performs better in smaller samples [20]. Only items with factor loadings of 0.40 and above were retained.

Confirmatory factor analysis (CFA) using IBM AMOS version 28 (Armonk, NY: IBM Corp.) was performed to verify the structural validity of the final version of HOPE-CKQ. The following model fit indices were used to determine adequacy of fit: chi-square goodness of fit should not be significant; comparative fit index (CFI) of more than 0.90 was acceptable and more than 0.95 was good; root mean square error of approximation (RMSEA) of less than 0.06 is indicative of a good fit, whereas a standardized root mean square residual (SRMSR) of less than 0.08 is indicative of an acceptable fit [25]. Internal consistency reliability was determined using Cronbach's alpha coefficient. A Cronbach alpha coefficient of more than 0.70 was good [22].

Known-groups validity testing was done by testing the hypothesis that caregiving knowledge was associated with parents’ formal education level. This was based on previous studies that showed an association between caregiving knowledge and parents’ level of education [10,26]. The scores of the HOPE-CKQ were compared across different levels of parents’ education using independent samples t-test, with the hypothesis that HOPE-CKQ scores would be significantly higher for parents with higher educational attainment, compared to parents with lower educational attainment. Hedges’ g coefficient was used to determine the effect size. Missing items were excluded list-wise.

Ethical approval to conduct this study was obtained from both the Institutional Medical Research Ethics Committees Ministry of Health Malaysia (ethical approval #NMRR-21-613-58896) and Universiti Kebangsaan Malaysia (ethical approval #UKM PPI/111/8/JEP-2021-413).

## Results

Phase 1: content validity index 

The I-CVI and S-CVI/Ave were calculated from scores submitted by the six evaluators. The final I-CVI for the 60-item HOPE-CKQ ranged from 0.83 to 1.00. The S-CVI/Ave for the 60-item HOPE-CKQ was 0.99. This met the criteria for good content validity. The evaluators agreed that the items were relevant to caregiving knowledge that were essential for parents.

Phase 2: the psychometric validation of HOPE-CKQ

A total of 167 completed responses were analyzed for factor analysis. Of these, 33.5% were obtained from the online survey and the rest were from face-to-face data collection. Table 1 displays the sociodemographic characteristics of the parents who participated in this study whereas Table 2 shows selected clinical characteristics of their child. Most respondents were female, of Malay ethnicity, from the lower income group, and had completed secondary education. Most of the children were diagnosed with leukemia (86.2%) before they started schooling (64.7%). About 40.7% of the children were still undergoing treatment at the time of recruitment.

**Table 1:** Sociodemographic characteristics of parents who participated in the study. *Parents’ education level was recategorized into secondary education level and tertiary education level for the present analysis. Primary and secondary education levels were collapsed into a single category because there were extremely few parents with only primary-level education. This was likely to be due to local policy for compulsory education up to secondary level.

Sociodemographic characteristics of the parents		n (%)	Mean (SD)
Age (years)		-	40.1 (7.8)
Gender	Male	39 (23.4)	-
	Female	128 (76.6)	-
Ethnicity	Malay	134 (80.2)	-
	Chinese	9 (5.4)	-
	Indian	9 (5.4)	-
	Others	15 (9.0)	-
Income	Lower (B40)	102 (61.1)	-
	Middle (M40)	50 (31.7)	-
	Upper (T20)	11 (6.6)	-
	Missing	1 (0.6)	-
Education^*^	Primary	4 (2.4)	-
	Secondary	71 (42.5)	-
	Tertiary	57 (34.1)	-
	Missing	35 (21.0)	-

**Table 2:** Clinical characteristics of the study respondents' children.

Clinical characteristics of the child		n (%)	Mean (SD)
Child’s age at diagnosis (years)		-	5.5 (3.7)
Preschool and younger (0-6 years)		108 (64.7)	-
School children (7-12 years)		48 (28.7)	-
Adolescent (12-18 years)		10 (6.0)	-
Missing		1 (0.6)	-
Diagnosis	Leukemia	144 (86.2)	-
	Lymphoma	23 (13.8)	-
Duration of diagnosis (years)		-	3.8 (4.2)
Up to two years		81 (49.1)	-
More than two years		81 (48.5)	-
Missing		4 (2.4)	-
Treatment phase	Receiving treatment	68 (40.7)	-
	Completed treatment	99 (59.3)	-

Exploratory Factor Analysis

Following the removal of items that did not meet the criteria for univariate normality and linearity, only 27 items were retained. These 27 items were subjected to preliminary internal consistency reliability test. Cronbach's alpha coefficient was 0.82 for the 27-item HOPE-CKQ. Removing two items with poor item-total correlations improved Cronbach's alpha coefficient to 0.83. Therefore, only 25 items were retained for EFA.

The Kaiser-Meyer-Olkin measure of sampling adequacy was 0.78 and Bartlett’s test of sphericity was statistically significant (chi-square=838.8, p<0.001), indicating that the sample was adequate for EFA. Parallel analysis using minimum rank factor analysis showed that only one factor should be retained, based on the 95% threshold for polychoric correlation [27]. This factor had an Eigenvalue of 6.97 and explained up to 27.9% of the variance. Table 3 shows the item factor loading and communalities for the 25 items. We retained items with factor loadings of 0.40 and above. This resulted in the 18-item version of the HOPE-CKQ.

**Table 3:** Factor loadings, communalities, and final item-total correlations from exploratory factor analysis of HOPE-CKQ. *Items were removed for further analysis.

No.	Item	Factor loading	Communality	Final item-total correlations
1b	Leukemia and lymphoma only happen when there are family members who have it	0.720	0.519	0.500
9c	Herbal supplements do not have any side effects	0.660	0.435	0.464
2c	Chemotherapy medication is only given intravenously (drip)	0.648	0.420	0.487
19a	Your child must remain lying down while the medication is given	0.628	0.395	0.381
17b	Alternative treatments can be given alongside chemotherapy	0.600	0.359	0.388
8c	Visitors who are ill may visit the child if they wear face masks	0.576	0.332	0.361
14a	It is not necessary to let your child know about his/her diagnosis because he/she will not understand	0.567	0.321	0.379
3a	Bruises on the skin are not a cause for concern	0.540	0.292	0.380
13a	Valid information is intended to promote certain products	0.539	0.290	0.364
18c	Cultured drinks containing probiotics are good for building your child's immune system during his/her treatment period	0.525	0.276	0.361
2b	Your child's treatment protocol should be the same as that of other children who have leukemia or lymphoma	0.524	0.275	0.374
7b	If your child’s mouth hurts, do not give him/her chilled foods	0.524	0.275	0.383
10c	Special complimentary milk may be given if recommended by a dietician	0.524	0.275	0.324
8b	Food that is kept overnight can be reheated to be given to the child	0.501	0.251	0.321
16c	Using the term "cancer" must be avoided in order to not frighten your child	0.490	0.240	0.336
9a	Supplements are not needed if your child eats a balanced diet	0.483	0.233	0.341
12b	All of your child’s medications must be kept in the refrigerator	0.447	0.199	0.310
12c	Your child’s medications must be kept together with your family’s medications	0.412	0.170	0.353
17a^*^	Alternative treatments are effective if there are testimonials of people being cured by that treatment	0.377	0.142	-
13c^*^	Information is valid if it is supported only by consumers’ testimonials	0.361	0.130	-
14c^*^	Avoid discussing the disease with your child to prevent them from feeling stressed	0.353	0.125	-
17c^*^	Alternative treatments need to be discussed with the doctor first before they can be given to your child	0.337	0.114	-
1c^*^	Leukemia and lymphoma patients need to undergo bone marrow examination	0.304	0.092	-
20b^*^	If your child does not want to eat his/her favorite food, you need to contact the hospital immediately	0.267	0.071	-
16a^*^	Tell your child that his/her illness is caused by a virus	0.208	0.043	-

Confirmatory Factor Analysis

**Figure 1:**
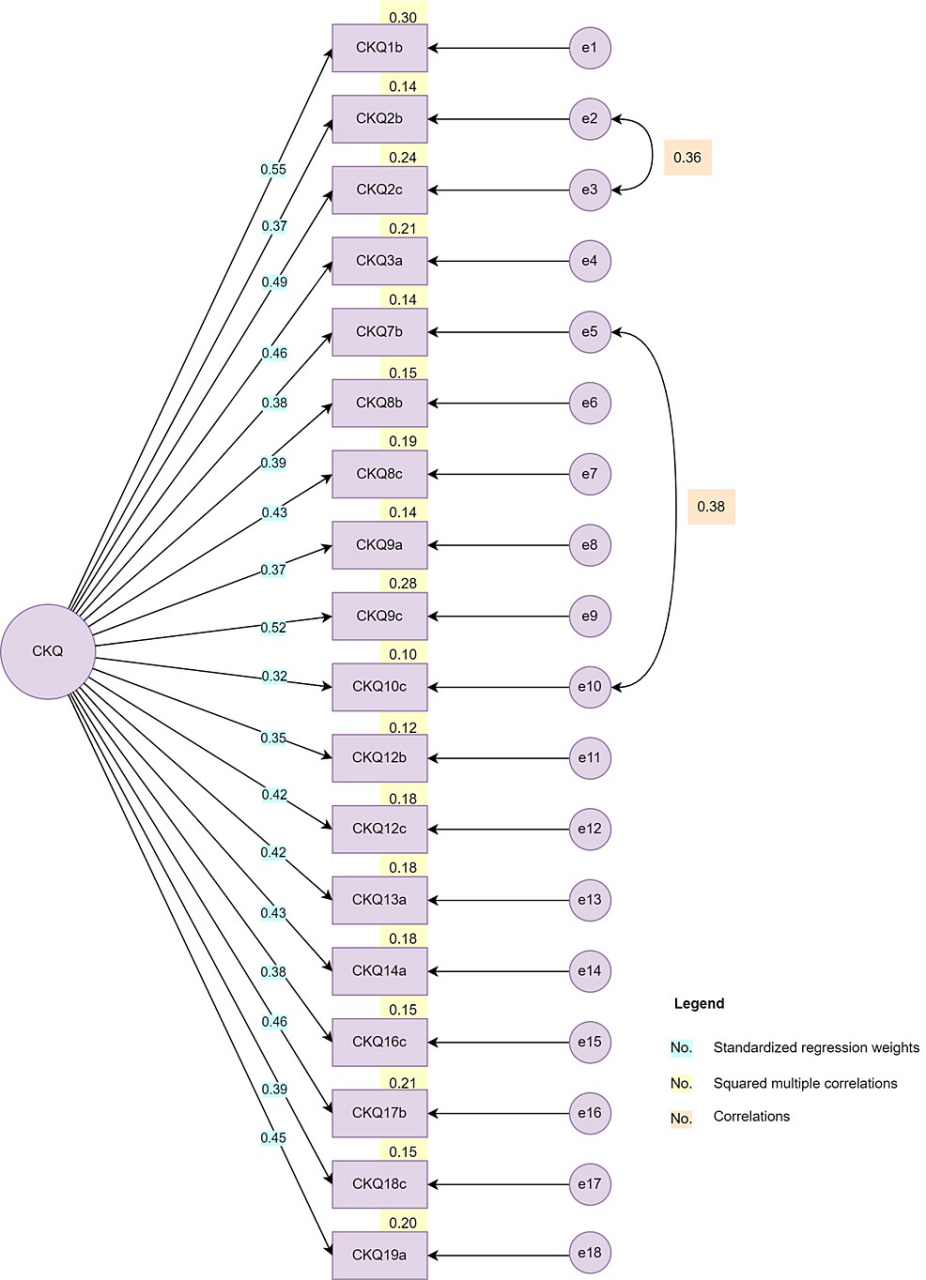
Eighteen-item HOPE-CKQ measurement model from confirmatory factor analysis. HOPE-CKQ: Hematological Oncology Parents Education Caregiving Knowledge Questionnaire

The 18-item HOPE-CKQ version was subjected to CFA. Figure 1 shows the 18-item HOPE-CKQ measurement model. Initial model fit indices showed statistically significant chi-square goodness of fit (chi-square=197.55, df=136, p<0.001), CFI of 0.839, RMSEA of 0.053, and SRMSR of 0.071. By allowing two pairs of error terms to covary (e2 and e3, e5 and e10), the model fit indices were improved as follows: the chi-square goodness of fit was no longer statistically significant (chi-square=153.22, df=133, p=0.111), CFI=0.95, RMSEA=0.030, and SRMSR=0.064. Fitting the one-factor 18-item HOPE-CKQ model to the present sample provided a good fit to the data. Evidence for structural validity of the HOPE-CKQ was established in the present study. The internal consistency of this unidimensional model is good with Cronbach's alpha of 0.80.

Reliability and Known-Group Validity for the 18-Item HOPE-CKQ

The internal consistency of this unidimensional model was good (α=0.80). The overall mean score for the HOPE-CKQ in this sample was 11.99 (SD 3.79). Parents with secondary level education had lower caregiving knowledge (mean score 10.33, SD 3.80) compared to parents with tertiary level education (mean score 12.61, SD 3.37), t=3.581, p<0.001. The Hedges’ g coefficient was 0.626 (95% confidence interval 0.27-0.98) indicating a medium effect size. Evidence for known group validity of the HOPE-CKQ was established in the present study.

## Discussion

A valid and reliable tool is needed to measure caregiving knowledge among parents of children with leukemia and lymphoma. This study described the development and validation of the HOPE-CKQ, an 18-item Malay language tool for such purposes. The HOPE-CKQ possessed content validity, structural validity, internal consistency reliability, and known-group validity.

Content validity refers to the degree to which the content of an instrument adequately reflects the construct to be measured [15]. Caregiving requires a broad range of knowledge that enables the parent to understand the child’s condition and to support the treatment process [8,9]. It is also important to evaluate parents’ knowledge about appropriate medical caregiving for the child. Inclusion of concepts such as alternative treatment in this tool reflected that parents often were faced with the decision of whether to give alternative treatment to their child [6,28]. In a Southeast Asian country such as Malaysia, it is important to evaluate parents’ knowledge about how to appraise information related to alternative treatment as part of their caregiving tasks. Another common caregiving task is to communicate the diagnosis with their child [29]. Appropriate content experts comprising pediatricians, family doctors, a nurse, and a parent were selected to rate the relevance of the items to caregiving knowledge. The content validity index for the HOPE-CKQ reflected the endorsement by content experts that the items reflected caregiving knowledge.

Construct validity of an instrument refers to the degree that the scores of the instrument are consistent with hypotheses, either through structural validity or by demonstrating differences between relevant groups [15,30]. For structural validity, the unidimensional structure of the HOPE-CKQ was supported by the results of exploratory factor analysis and confirmatory factor analysis. The use of EFA with polychoric correlation provides a more robust factor solution for a scale with binary scores [19,21]. This is because conventional EFA using the Pearson correlation matrix tends to underestimate the correlations between binary or ordinal scales [19]. The HOPE-CKQ had good internal consistency, with Cronbach's alpha of 0.80. The known-group validity method was used to test the hypothesis that the scores of the HOPE-CKQ were associated with parents’ education level [10,26]. Parents with tertiary education levels had higher caregiving knowledge compared to parents with secondary education levels. This established the known-group validity of the HOPE-CKQ and its ability to discriminate between higher and lower levels of caregiving knowledge.

The HOPE-CKQ questionnaire has items that assess general information about disease and treatment (items 1b, 2b, 2c), medical caregiving (items 3a, 7b, 8b, 8c, 10c, 12b, 12c, 18c, 19a), alternative treatment literacy (9a, 9c, 13a, 17b), and communication with the child (14a, 16c). This suggests the presence of a second-order measurement model. Future research may extend the present study by validating the proposed factor structure.

Study strengths and limitations

To the best of our knowledge, the HOPE-CKQ is the first validated and reliable scale to measure caregiving knowledge among parents of children with leukemia and lymphoma. The present study has some limitations to be addressed in future research. Firstly, the scale was administered in the Malay language and will require linguistic validation for cross-cultural use. Secondly, to counteract the issue of overfitting, separate samples should be used for EFA and CFA of the HOPE-CKQ. Therefore, CFA using a separate set of data in the future will be required to demonstrate the reproducibility of the model fit. Last but not least, the scale’s test-retest reliability was not tested in the present study. Future studies could address this limitation by asking participants to complete a second HOPE-CKQ assessment about one to two months after the first.

## Conclusions

The HOPE-CKQ was found to be a valid and reliable tool to measure caregiving knowledge among Malaysian parents of pediatric leukemia and lymphoma patients. The utility of this scale has yet to be evaluated in the context of prevention and intervention programs concerning parental caregiving knowledge. The HOPE-CKQ may be considered to measure caregiving knowledge as an outcome for prevention and intervention programs in the future. It is brief, easy to administer, and encompasses various relevant domains. Despite several limitations, it has value for future research on caregiving knowledge among parents of pediatric leukemia and lymphoma patients.
